# Is palindromic rheumatism amongst children a benign disease?

**DOI:** 10.1186/s12969-018-0227-z

**Published:** 2018-02-13

**Authors:** Yonatan Butbul-Aviel, Yosef Uziel, Nofar Hezkelo, Riva Brik, Gil Amarilyo

**Affiliations:** 1 Department of Pediatrics B, Ruth Rappaport Children’s Hospital, Rambam Medical Center, Haifa, Israel; 2 Pediatric Rheumatology Service, Ruth Rappaport Children’s Hospital, Rambam Medical Center, Haifa, Israel; 3 The Ruth and Bruce Rappaport Faculty of Medicine, The Technion - Israel Institute of Technology, Haifa, Israel; 4 Sackler Faculty of Medicine, Tel Aviv University, Ramat Aviv, Israel; 5 Pediatric Rheumatology Unit, Department of Pediatrics, Meir Medical Center, Kfar Saba, Israel; 6 Pediatric Rheumatology Unit, Schneider Children’s Medical Center of Israel, 14 Kaplan St., 4920235 Petach Tikva, Israel

**Keywords:** Palindromic rheumatism, Children, Juvenile, Idiopathic, Arthritis, Palindromic

## Abstract

**Background:**

Palindromic rheumatism is an idiopathic, periodic arthritis characterized by multiple, transient, recurring episodes. Palindromic rheumatism is well-characterized in adults, but has never been reported in pediatric populations. The aim of this study was to characterize the clinical features and outcomes of a series of pediatric patients with palindromic rheumatism.

**Methods:**

We defined clinical criteria for palindromic rheumatism and reviewed all clinical visits in three Pediatric Rheumatology centers in Israel from 2006through 2015, to identify patients with the disease. We collected retrospective clinical and laboratory data on patients who fulfilled the criteria, and reviewed their medical records in order to determine the proportion of patients who had developed juvenile idiopathic arthritis.

**Results:**

Overall, 10 patients were identified. Their mean age at diagnosis was 8.3 ± 4.5 years and the average follow-up was 3.8 ± 2.7 years. The mean duration of attacks was 12.2 ± 8.4 days. The most frequently involved joints were knees. Patients tested positive for rheumatoid factor in 20% of cases.

One patient developed polyarticular juvenile idiopathic arthritis after three years of follow-up, six patients (60%) continued to have attacks at their last follow-up and only three children (30%) achieved long-term remission.

**Conclusions:**

Progression to juvenile idiopathic arthritis is rare amongst children with palindromic rheumatism and most patients continued to have attacks at their last follow-up. Longer follow-up periods are required to predict the long-term outcomes of pediatric patients with palindromic rheumatism.

## Background

Palindromic rheumatism (PR), originally described in 1944 by Hench and Rosenberg, is an idiopathic, periodic arthritis characterized by multiple, transient, recurring episodes of mono- or oligo-arthritis, associated with tissue swelling around the involved joints [1]. Episodes last from a few hours to a few days and then resolve spontaneously. Between episodes, there is no residual joint damage.

The condition involves little or no constitutional symptoms or abnormalities in laboratory tests, and no significant functional, pathologic, or radiological changes, even after years of disease.

In 1986, Pasero and Barbieri proposed five criteria for the diagnosis of PR, which include: (1) at least a six-month history of brief, recurrent episodes of mono-arthritis or polyarthritis with sudden onset, (2) direct observation by a physician of at least one attack, (3) involvement of three or more joints, although they could be involved at different times, (4) complete absence of radiographic findings, and (5) exclusion of all other arthritides [2].

Estimated prevalence is 1/8 to 1/20 compared to rheumatoid arthritis (RA) cases [2, 3]. Female predominance is similar to that observed among RA patients [4, 5], and cases were reported in persons 20 to 80 years-of-age [4]. Except for one recent case report, no cases have been described to date in the pediatric population [5]. There is no known ethnic predisposition to the disease.

The differential diagnosis of PR is broad and includes rheumatoid arthritis; crystalline arthropathies, such as gout; seronegative spondyloarthropathies, such as reactive arthritis or inflammatory bowel disease-related arthritis; Whipple’s disease; Behcet’s syndrome; relapsing polychondritis; intermittent hydrarthrosis and familial Mediterranean fever (FMF).

The aim of this study was to describe the clinical and laboratory features, therapies and follow-up outcomes of a series of pediatric patients with PR.

## Methods

This cross-sectional, retrospective study was based on a review of medical records from patients referred to three, large, tertiary academic hospitals in Israel. To identify cases, a preliminary search of the electronic medical records of the outpatient pediatric rheumatology clinics (run by the authors YB, RB, YU, GA) was conducted. The search included the words arthropathy OR juvenile OR arthritis in the field “diagnosis” during the 10-year period from March 2006 to May 2016. The study was approved by the local Institutional Review Board of each participating hospital. Informed consent was waived.

Inclusion criteria were based on the Pasero and Barbieri criteria with some modifications, and included: age 1–16 years at presentation, recurrent attacks (at least three) of sudden-onset monoarthritis, polyarthritis or periarticular tissue inflammation lasting from a few hours to 5 weeks, verification of at least one attack by a physician and exclusion of all other forms of arthritides [2].

Retrieved data included demographic characteristics, age at onset of the disease, family history, attack frequency, triggering factors, duration of attacks, involved joints, and symptoms between attacks (if any). Laboratory tests included complete blood count, erythrocyte sedimentation rate (ESR), C-reactive protein (CRP), rheumatoid factor (RF), anti-CCP, anti-nuclear antibody (ANA), anti-double stranded DNA antibody (anti-dsDNA) and imaging data. When available, results from other diagnostic tests, such as the pathergy test, human leukocyte antigen (HLA)-B27/B5 test, radiography of other parts, genetics for common MEFV mutations and ophthalmologic examination data were collected.

## Results

Among 630 records identified, 11 matched the inclusion criteria and were reviewed thoroughly. One patient was excluded due to sparse follow-up data. Ten pediatric patients with palindromic rheumatism were diagnosed from March 2006 to May 2016. Demographic characteristics of the patients are presented in Table 1.

**Table 1:** Demographic data of pediatric patients with palindromic rheumatism

Age at diagnosis (years ± SD)	8.3 ± 4.5
Male/female (N)	6/4
Number of episodes during follow up **(**N ± SD); (range)	11.3 ± 8.8; (4–29)
Length of follow-up ^a^ (years ± SD)	3.8 ± 2.7
Total number of involved joints (no. ± SD)	3.1 ± 1.6
Systemic symptoms during attacks (%)	20
Average duration of episodes(days)	12.2 ± 8 .4
Morning stiffness during attacks (%)	50

^a^Defined as the time from first presentation to last follow-up

Five patients (50%) had a family history of rheumatic disease, including two with a family history of Crohn’s disease, one of RA and two patients of psoriasis.

The duration of follow-up ranged from 6 months to 9.9 years (median 3.5 years) and the number of episodes ranged from 3 to 29 (median 9.5 episodes).

Except for arthritis, no patients had any other systemic manifestations (including fevers) during flares. The knee was the joint most frequently involved and all patients had knee arthritis at least once. The second most frequently involved joints were the wrists and ankles in three patients, each (30%) (Table 2). In nine patients (90%), flares were monoarticular or oligoarticular.

**Table 2:** Prevalence of involved joints during attacks in pediatric patients with palindromic rheumatism

Joints	Percentage
Knees	100
Wrists	30
Ankles	30
Hips	20
Elbows	10
Fingers	10

### Laboratory and radiographic findings

During flares, C-reactive protein (CRP) was elevated in four patients (40%), the average value was 21.5 mg/dl (median 2.5 mg/dl, range 0.5–120 mg/dl). The erythrocyte sedimentation rate (ESR) measured in eight patients was higher than 30 mm/h in half of the cases. No leukocytosis was noted in any patient.

RF was measured in nine patients and was positive, albeit in low titers, in two patients (22%). Antinuclear antibodies (ANA) were measured in nine patients and were positive (defined as a titer higher than 1:80) in three patients (33%). HLAB27 antigen was measured in three patients and was negative in all cases.

Genetics for the most common MEFV mutations were evaluated in six patients, none of whom was found to be a carrier. The affected joints were x-rayed at least once in eight patients; no erosions, joint space narrowing, or subchondral cyst erosions were observed. One patient had a bone scan that showed evidence of arthritis. Two patients had US of the affected joint (2 knees and 1 elbow) during flare, which demonstrated evidence of synovitis with variable degrees of synovial effusion. Six patients were examined by an ophthalmologist and had normal results.

Six children had joint aspiration. Only one had a markedly elevated synovial white blood cell count (57.300 × 10^9^/l). Synovial fluid cultures were negative in all cases.

### Medications and long-term follow-up

Three patients were treated during flares with a short course of systemic corticosteroids, all with good response. One patient received a local corticosteroid joint injection with flare resolution.

A trial of a prophylactic agent was administered to six patients: colchicine to four with partial response, and two patients were treated with NSAIDS for long periods with no response.

Patients were asymptomatic between flares, and only one developed fever during a portion of the episodes.

Long-term remission was defined as at least six months with no flares. and was achieved in three patients (30%) during the follow-up period. These included one who was treated with prophylactic colchicine, but eventually developed JIA.

In addition, one patient developed polyarticular JIA 3 years after experiencing palindromic flares in his knee and wrists. Six patients (60%) continued to have attacks and never achieved long-term remission (followed for 5.3 ± 2.7 years).

## Discussion

Although PR is well-described in the adult population, to the best of our knowledge it has never been reported in the pediatric population, except for one recent case report [5]. The definition of PR in adults is not well-established and a few criteria have been suggested. In 1987, Hannonen et al. [2] suggested criteria that included: (1) recurrent attacks of sudden-onset monoarthritis, polyarthritis or periarticular tissue inflammation, lasting from a few hours to one week; 2) verification of at least one attack by a physician; 3) subsequent attacks in at least three different joints; and 4) exclusion of other forms of arthritides [6]. This set of criteria has not been adopted by the American Rheumatism Association or by the American College of Rheumatology.

The criteria used in this study were based on the Hannonen criteria [6], as in our experience, some of the attacks lasted longer than one week. The length of episodes in most of the patients ranged from a few days to two weeks. The first presenting episode ranged from 2 days to 2 weeks.

Unlike JIA, which affects mostly females, men were more commonly affected in our series. The literature provides mixed information as to whether there is a sex difference in adult PR, and a report which suggests male predominance [7].

In terms of joints affected, knee, wrists, and small joints of the hand have been reported in the literature as most commonly affected in adult PR. In our series, most of the patients experienced involvement of the large joints and all had knee arthritis at least once [3].

Unlike in the adult population, the differential diagnosis of pediatric PR may include a number of unique clinical entities, such as sport and playground injuries; joint hypermobility/Ehlers-Danlos; transient hip synovitis; post-infectious arthritis, (such as post streptococcal reactive arthritis); osteochondrosis, and pain amplification syndromes. These possibilities, as well as others were carefully ruled out by the treating pediatric rheumatologists after extensive workup.

Family history of rheumatic disease was relatively common in our series (45%), and most of the reported family diseases were of psoriasis and inflammatory bowel disease. This finding has not been reported previously.

Cañete, et al. found a high frequency of MEFV mutations in Spanish PR patients with anti-citrullinated protein antibody-negative PR, compared with those with positive anti-citrullinated protein antibody (22.2% vs. 5.3%) [8]. FMF is not a rare disease in the Israel population, and although PR is not a typical presentation of FMF, six patients had negative tests for the common MEFV mutation. Four patients were treated with colchicine and all exhibited only partial response.

Inflammatory markers were elevated in 40% of our patients (40% to 50%), comparable to adult PR. RF is usually found in 40% to 65% of adult PR, and its presence has been associated with a more severe form of the disease, as well as with a higher probability of eventually developing classic RA [6]. In our series, only two patients (ages 9 and 15) had low, positive RF titers, and anti-CCP levels were not examined in these patients.

Although most of the patients in our series did not achieve long-term remission, only one child developed JIA after 3 years of PR. In adults, it has been reported that at one-year follow-up, 27.5% of patients with PR developed RA and 3% developed systemic lupus erythematosus, while 50% of the patients developed different types of chronic arthritis [9]. The outcomes in our series were better than that in adults, but a longer follow-up period is required to determine the percentage of pediatric PR patients who will develop chronic arthritis. The literature reports only one case of progression of PR to JIA, that occurred in a Japanese girl carrying heterozygous L110P-E148Q substitutions of the MEFV gene [5].

Limitations of our study include its retrospective nature, the paucity of described cases and a short follow-up period of 0.5–9.9 years. Moreover, since FMF is relatively common in the Israeli population and the MEFV gene is not fully screened, some of our patients may have an unusual presentation of FMF. Nonetheless, Israeli rheumatologists are very familiar with FMF and in all cases where FMF was suspected, even with a negative genetic test, a colchicine trial was initiated. No patient displayed a prominent response to colchicine therapy.

## Conclusion

This is the first case series reported of a pediatric population with PR which summarizes clinical follow-up of 10 patients from 3 large Israeli tertiary pediatric medical centers. Progression to juvenile idiopathic arthritis in this children was found to be uncommon and most patients continued to have attacks at their last follow-up. Clinicians should be aware of this disease in children and it should be considered in the differential diagnosis of any child who presents with recurrent acute arthritis and negative work-up for autoimmune or autoinflammatory disease. Additional studies are needed to better describe PR and its long-term outcomes, and to determine optimal treatments for this syndrome.
